# Progression of obstructive sleep apnoea after renal denervation is not associated with hypertension exaggeration

**DOI:** 10.1186/s12890-023-02757-1

**Published:** 2023-11-23

**Authors:** Lyudmila S. Korostovtseva, Mikhail V. Ionov, Elizaveta A. Shcherbakova, Mikhail V. Bochkarev, Igor V. Emelyanov, Yulia S. Yudina, Svetlana A. Mironova, Dmitry A. Zverev, Dmitry S. Lebedev, Aleksandr D. Vakhrushev, Natalia G. Avdonina, Nadezhda E. Zvartau, Evgeny N. Mikhaylov, Yurii V. Sviryaev, Aleksandra O. Konradi

**Affiliations:** Almazov National Medical Research Centre, 2 Akkuratov str., St Petersburg, 197341 Russia

**Keywords:** Obstructive sleep apnea, Apnea-hypopnea index, Resistant hypertension, Renal denervation, Sympathetic nervous system

## Abstract

**Purpose:**

In a cohort, observational prospective trial, we assessed the long-term dynamics of sleep-disordered breathing in patients with resistant hypertension after renal denervation and their association with blood pressure change at remote follow-up.

**Materials and methods:**

Twenty-eight patients with stable hypertension who were recruited for endovascular radiofrequency renal denervation in 2012–2019 and had valid both baseline and follow-up sleep study, were included in the analysis. All patients underwent physical examination, anthropometry, office and ambulatory blood pressure measurements, blood and urine tests, kidney visualization, and full polysomnography before and within 12–36 months after renal denervation.

**Results:**

The average follow-up comprised 30.1 ± 8.4 months. At long-term follow-up, no significant changes in creatinine level, estimated glomerular filtration rate, body mass index were registered. There was a significant increase in sleep apnea severity indices: the mean change in apnea-hypopnea index comprised 9.0(-21.1;25.2) episodes/h, in oxygen desaturation index 6.5(-16.8;35.9) episodes/h, in the average SpO_2_ -1.7(-5.6;1.9)%. Over 12-month follow-up, there were no significant differences in blood pressure response in patients with and without sleep apnea. The baseline apnea-hypopnea and oxygen desaturation indices and the mean SpO_2_ were associated with the circadian blood pressure profile at follow-up, but did not correlate with the blood pressure response.

**Conclusions:**

Although the severity of sleep apnea worsens at > 12 months follow-up after renal denervation, this is not associated with hypertension exaggeration.

**Supplementary Information:**

The online version contains supplementary material available at 10.1186/s12890-023-02757-1.

## Introduction

Obstructive sleep apnea (OSA) characterized by the repetitive episodes of upper airway complete (apneas) or partial (hypopneas) collapse during sleep, is the most common type of sleep-disordered breathing (SDB) [1]. OSA is considered a risk factor for hypertension (HTN) and one of the main causes of resistance to antihypertensive therapy [2]. In clinical studies, OSA is diagnosed in more than two thirds of patients with resistant HTN [3].

Activation of sympathetic nervous system resulting from intermittent hypoxia and hypercapnia, altered chemoreflex, and repeated arousals, is considered a key mechanism underlying the association between OSA and blood pressure (BP) increase [4]. OSA-related BP elevation can be persistent both during nighttime and daytime and is frequently associated with the altered nocturnal BP dipping [5]. The effective therapy of OSA is associated with improved blood pressure (BP) control, although the data are controversial and the effect varies depending on multiple factors (i.e. initial BP values, patients’ compliance, presence of daytime symptoms, namely daytime sleepiness etc.). The “gold standard” treatment by continuous positive airway pressure (CPAP) therapy might reduce BP with the greater impact on nocturnal values leading to an improvement in nocturnal BP dipping [6–10]. Other therapies, i.e. oral appliances, demonstrate similar action although their efficiency [9] might be lower and the data are not sufficient. However, the estimated mean decrease does not exceed 2–4 mmHg for systolic and diastolic BP (SBP and DBP).

Renal denervation (RDN) was implemented as a treatment option in resistant HTN substantiated by the experimental and clinical evidence of the reduced central sympathetic activity [11] and, consequently, significant BP drop. In the last decade, RDN overcame the rise and fall, related to the promising results of the first trials SYMPLICITY HTN-1 and − 2 [12, 13] followed by a disclaimer due to the first negative results of the sham-controlled randomized SYMPLICITY HTN-3 Trial. The latter [14] failed to prove a significant antihypertensive effect of the RDN procedure. However, a recent analysis of the long-term data of SYMPLICITY HTN-3 Trial demonstrated a larger drop in BP and better BP control compared with patients who received sham procedure after 36 months post-procedure [15]. Novel technologies in RDN has been also shown to be safe and efficient both at short- and long-term. Ultrasound RDN showed to be safe and efficient at 2-month follow-up in a RADIANCE-HTN TRIO study which overcame limitations of previous RDN-related studies, including strictly controlled antihypertensive treatment, standardized surgery procedures, effective masking of both patients and medical staff etc. [16]. A sustained BP reduction up to 24–36 months after radiofrequency RDN was confirmed in the multicenter SPYRAL HTNON MED [17]. Following hot discussion, a balanced and cautious approach to the application of RDN was developed. Currently it is considered an interventional technology to treat resistant HTN and the efforts are turned to search the predictors of clinical response. A number of clinical features are considered important (i.e. baseline BP level and heart rate, abdominal obesity, plasma renin activity and aldosterone, heart rate variability, orthostatic hypertension, medication at baseline, renal artery anatomy etc.), and the clinical conditions associated with the sympathetic hyperactivity are of great concern [11, 18].

Due to the high sympathetic drive, OSA patients are considered a promising group who could benefit from RDN. Moreover, other protective effects of RDN are widely investigated including reduction in ventricular arrhythmias [19, 20], atrial fibrillation recurrence [21], improvement in renal function [22], glucose control [23], target organ damage, etc. [24].

In 2011, Witkowski et al. [25], in a small cohort, in an open, non-randomized study demonstrated a decrease in apnea-hypopnea index (AHI) after RDN, which was associated with the improvement in clinical manifestation of OSA and sustained BP reduction up to 6 months [26]. Since then, several other scientific groups investigated the effects of RDN on OSA severity. Several potential mechanisms which could mediate the beneficial effect of RDN on OSA are suggested and include a decrease in extracellular fluid volume due to higher salt excretion and nocturnal rostral fluid shift, central effects on respiratory center.

However, the available data are very limited and controversial, the persistence and clinical relevance of the AHI change are questionable, and the majority of the studies are limited by 3–6 months follow-up.

We aimed to test the hypothesis that the severity of SDB decreases at long-term follow-up post-RDN and this change is associated with the better BP response.

The objective of this study was to assess the long-term dynamics of SDB in patients with resistant HTN after RDN and their association with BP change at extended follow-up.

## Design and methods

In an open prospective single-center study, we included patients with suspected resistant HTN referred to the center at Almazov National Medical Research Center (St Petersburg, Russia) which is recognized as one of the ESH hypertension excellence centers. These patients were recruited for endovascular radiofrequency RDN in 2012–2019 as per inclusion/non-inclusion criteria.

Inclusion criteria were: age 20–65 years old; BP ≥ 140/90 mmHg despite regular intake of at least 3 antihypertensive drugs in appropriate doses including a diuretic, and informed consent.

Non-inclusion criteria were: secondary endocrine HTN; severe kidney dysfunction (estimated glomerular filtration rate (eGFR) < 45 ml/min/1.73m^2^); renal stenosis or anatomical features, e.g. diameter of the renal artery < 4 mm, fibromuscular dysplasia; renal angioplasty with stenting for renovascular HTN in the past; severe concomitant diseases (cardiovascular, urogenital, uncontrolled diabetes mellitus, manifesting thyroid disease, morbid obesity with body mass index (BMI) > 40 kg/m^2^, liver diseases, any oncological diseases in past 5 years, rheumatoid diseases, open surgery in the last year); drug use, alcohol abuse; pregnancy (or planned pregnancy), lactation; and cognitive decline, inability to follow the protocol. For this analysis, the available sleep study at baseline and follow-up was an additional inclusion criterion.

**Fig. 1:**
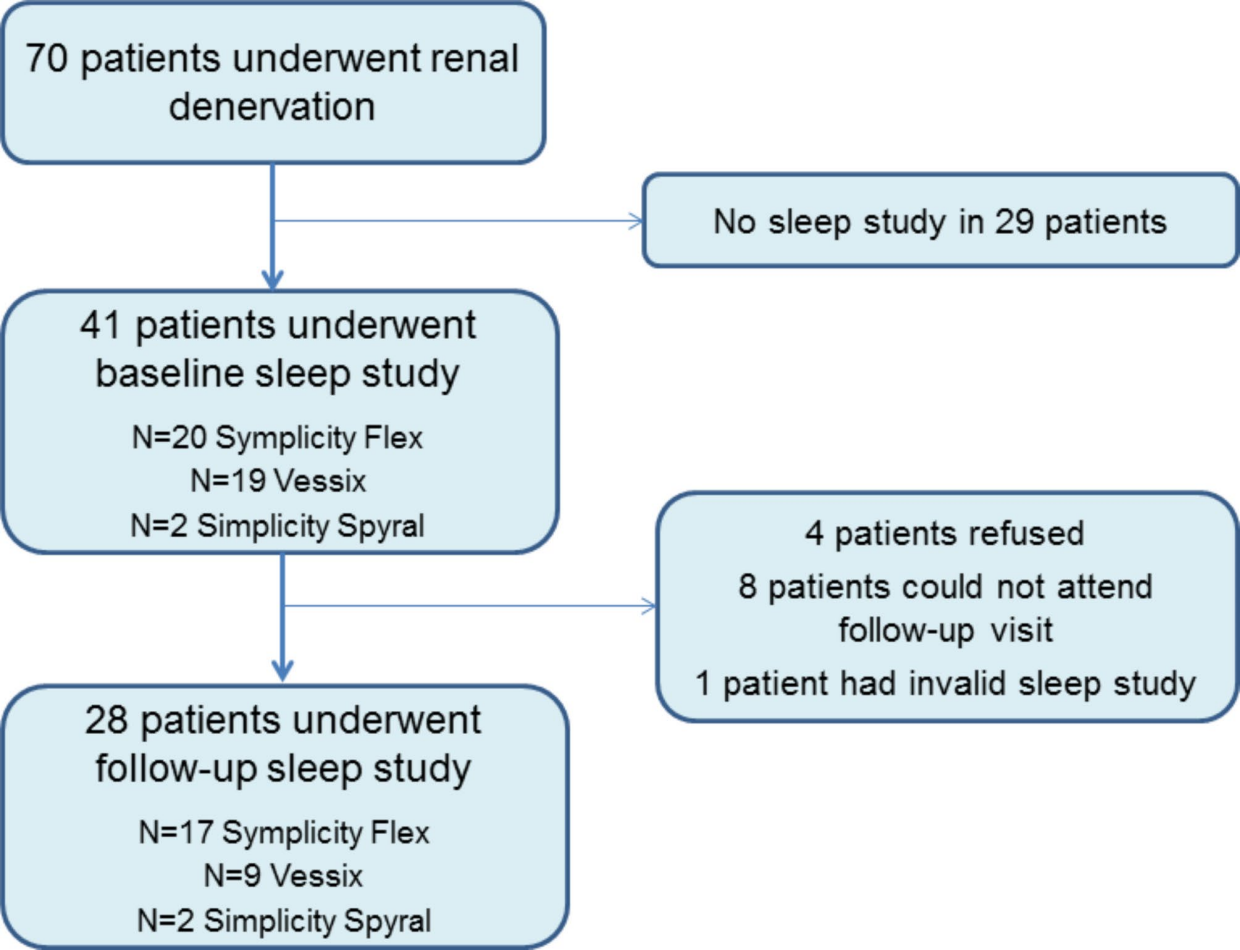
Study flow chart

Among 70 patients who underwent RDN, 41 patients had valid baseline sleep study, while 28 patients had both baseline and follow-up sleep studies (Fig. 1) and were included in the present analysis.

The study was performed in accordance with the principles of Good Clinical Practice and 2013 Declaration of Helsinki. The study protocol was approved by the Scientific Council of Almazov National Medical Research Centre on 23.12.2011 (protocol №11), and later the updated version of the protocol was approved in 2018 by the Local Ethics Committee of Almazov National Medical Research Centre (protocol №2018-12-11). All patients signed informed consent before the enrollment in the study.

## Examination

All patients underwent physical examination, anthropometry, office BP measurements, ambulatory blood pressure monitoring (ABPM), fasting blood tests for renal function assessment (creatinine, eGFR), lipids and glucose, urine for microalbuminuria, renal Doppler ultrasonography (Vivid-7, General Electric, USA), renal CT scan (Magnetom Tria aTim 3 T Siemens, Germany), and sleep study.

### Anthropometry

Among anthropometric measurements, we assessed height (accuracy up to 0.5 cm), weight (accuracy up to 100 g), and waist and hip circumferences (in cm) and calculated body mass index (BMI): weight (kg)/height^2^ (m^2^) (the Quetlet equation). Obesity was diagnosed in case of BMI ≥ 30 kg/m^2^.

### Office BP measurements

Office BP measurements were performed at all visits according to the guidelines on the management of HTN of the European Society of Cardiology/European Society of Hypertension (ESC/ESH) [2]: after 5 min of rest in a sitting position, three measurements with 1–2 min intervals were performed on the right hand (the automatic tonometer Omron M3 Expert [HEM 7132-ALRU], Japan). The average of the last two BP readings was taken for the analysis.

### Ambulatory BP monitoring

Ambulatory BP monitoring (ABPM) was performed with the use of a certified oscillometric device (BPLab, “Piotr Telegin” LLC, Russian Federation). The measurements were performed every 15 min during the day (08:00–22:00), and every 30 min during night (22:00–08:00). The nighttime period was later corrected according to the patient’s diary. The data were considered valid when at least 70% measurements were successful. We assessed average 24-hour BP, daytime and nighttime BP, daytime and nighttime BP load (%), circadian BP profile. According to the ESC/ESH guidelines the following diagnostic thresholds were considered: mean 24-hour BP ≥ 130/80 mm Hg; mean daytime BP ≥ 135/85 mm Hg; and mean nighttime BP ≥ 120/70 mm Hg. Circadian BP profile was assessed separately for systolic and diastolic BP by the equation: 100×(mean BPday-mean BPnight)/mean BPday (values ≥ 10% indicate dippers, while values < 10% indicate non-dippers). For quantitative analysis a circadian coefficient was calculated by formular mean BPnight/mean BPday (values ≥ 0.9 indicate non-dipper profile, while values ≤ 0,8 identify dippers).

### Blood tests

The following blood tests were assessed in a fasting state: lipids, serum creatinine, glucose, and insulin (Cobas e411 and Cobas Integra 400 plus, Switzerland; reagents from Roche-diagnostics, Germany). The lipid panel included the total cholesterol (TC), low-density lipoproteins (LDL). Dyslipidemia was diagnosed when the total cholesterol was 6.0 mmol/l and higher and/or LDL was 3.0 mmol/L and higher or in case of the intake of hypolipidemic drugs [27]. Impaired fasting glucose was diagnosed when the fasting glucose exceeded 5.6 mmol/L; diabetes mellitus was diagnosed either based on previous medical history or if the fasting glucose level equals 7.0 mmol/L [28] or more [27]. The glomerular filtration rate was estimated by the 2009 CKD-EPI equation.

### Sleep study

In-hospital unattended full polysomnography (PSG) was performed at cardiology department before RDN and at 1-year follow-up. The recordings included the following traces: electroencephalogram, electrooculogram, electromyogram of chin muscles, electrocardiogram, oronasal airflow (via both nasal cannulas and thermistor), pulseoximeter, snoring, thoracic and abdominal respiratory movements, and body position. The baseline and follow-up recordings were scored manually by experienced specialists (LK, MB) according to the Scoring rules of the American Academy of Sleep Medicine version 2.5 [29].

None of the patients with SDB used non-invasive ventilation (due to the lack of reimbursement of CPAP-therapy in Russia most of the patients with SDB denied therapy, two patients did not tolerate the device).

### Renal denervation procedure

All patients underwent renal angiography via Seldinger technique followed by bilateral RDN. Three specialized endovascular ablation systems were used: Symplicity flex™ (Medtronic Inc, Mountain View, Canada) in 22 patients, Vessix™ Renal Denervation System (Boston Scientific, USA) in 27 patients, and Symplicity Spyral™ Renal Denervation system (Medtronic, USA) in 21 patients.

By the Symplicity flex™ (Medtronic Inc, Mountain View, Canada) radiofrequency ablations were applied from distant to proximal part of the renal arteries under electrode temperature control (40–75°С), with the maximal output power of 8 W. The maximum of 8 applications were performed at > 5 mm distance in a spiral pattern, each lasting for about 120 s with the total duration of the procedure up to 35-45-minutes.

In the Vessix™ Renal Denervation System, balloon catheters with 4-, 5- and 6-mm diameter depending on the diameter of renal artery were used. Radiofrequency generator monitored impedance, and in case of the inadequate electrode contact, the electrode switched off automatically. Each application lasted for 30 s, temperature 65–68 °С, power 1 W.

The Symplicity Spyral™ device monitors temperature and impedance and regulates power (6 W), duration of applications is 60 s. All procedures were performed by well experienced and properly trained operators (DZ, EM).

Upon completion of the procedure, femoral artery was compressed. For 12 h the patients were monitored at the intense care unit followed by 2-5-day stay at the cardiology department. Two-month dual antiplatelet therapy (aspirin 100 mg and clopidogrel 75 mg OD) was prescribed to all patients.

Acute kidney injury (contrast-induced nephropathy) was diagnosed according to KDIGO criteria [30], mainly if serum creatinine increased by ≥ 26.5 µmol/l within 48 h after RDN.

### Follow-up and outcomes

The follow-up visits were performed regularly at 3, 6, 12, 24, 36 months. In this analysis, the long-term follow-up data (> 1 year) are presented.

At follow-up visits clinical investigation included physical examination, anthropometry, office BP measurements, ABPM, blood tests. Follow-up sleep study (full polysomnography at the sleep lab) was performed within 12–36 months after RDN. Antihypertensive therapy was re-evaluated and modified if needed (upon the discretion of investigator on case-by-case basis), but not earlier than 1 month after the procedure.

In this analysis, the primary outcome was the mean change in the main indices of SDB severity (AHI, ODI, SpO_2_ levels) from baseline to the follow-up assessment by PSG. We also assessed the association between change in sleep apnea parameters and BP change. The BP response was evaluated as the decrease of BP by at least 5 mmHg from the baseline (ΔBP = BP_follow−up_ – BP_baseline_).

### Statistical analysis

Descriptive statistics included the mean and standard deviation values for the normally distributed variables and median (minimum-maximum) for non-normally distributed variables. Due to the sample size < 50, the Shapiro-Wilk test was applied to assess the distribution.

We applied a frequency analysis (the chi-square) to assess the contingency between the nominal and categorical variables. We applied MacNemar test for the paired nominal variables. The continuous variables were compared by Mann-Whitney U test, and for paired data we applied Wilcoxon signed-rank test. Spearman’s rank correlation coefficient was used to assess the association between the parameters of interest. Logistic regression analysis was used to assess the associations between circadian BP profile status (as the dependent categorical variable, dipping/non-dipping) and indices of AHI severity – AHI, ODI, mean SpO_2_ (included separately as independent variables), with the adjustments for other potential confounders (age, sex, BMI). The results for the estimated potential predictors are presented as the odds ratio (OR) and 95% confidence interval (95% CI). The two-sided *p*-value < 0.05 (in case of multiple testing for 3 groups < 0.005) was accepted to define the validity of the statistical hypothesis. The software SPSS 20.0 was used to perform the statistical analysis.

## Results

### Patient characteristics at baseline

Out of forty-one patient with baseline sleep study, twenty-eight patients (including 16 patients with SDB at baseline) underwent sleep study at follow-up (Table 1): four patients denied the study, 8 patients could not attend the sleep lab, and in one patient the study was not valid (Fig. 1). Those with and without sleep studies at baseline and at follow-up were matched by the main clinical parameters (Suppl. Table  1). Although included patients (*n* = 28) had higher values of the baseline office BP compared to the patients not included in the analysis (*n* = 42), the ABPM data were comparable in these subgroups. Otherwise, they did not differ by the main clinical and instrumental parameters.

**Table 1:** The baseline characteristics of patients who underwent baseline and follow-up sleep study

Parameter	Total (*n* = 28) median (min; max)	With SDB (*n* = 16)	Without SDB (*n* = 12)	*p*-value
Age, years	54.5 (27; 69)	56.5 (46; 69)	47 (27; 64)	*p* = 0.057
Sex (male), *n* (%)	15 (54%)	9 (56%)	6 (50%)	χ^2^ = 0.32, p = 0.57
Hypertension duration, years	16.5 (4; 36)	18 (4; 33)	18 (6; 35)	*p* = 0.76
**Number of antihypertensive drugs**	**4 (2; 7)**	**4.5 (3; 7)**	**4 (2; 5)**	***p*** ** = 0.049**
BMI, kg/m^2^	30.0 (24.2; 44.2)	30.2 (24.2; 44.2)	30.0 (26.4; 33.6)	*p* = 0.76
Obesity, *n* (%)	15 (54%)	9 (56%)	6 (50%)	χ^2^ = 0.26, *p* = 0.61
Type 2 diabetes mellitus, *n* (%)	7 (25%)	5 (31%)	2 (17%)	χ^2^ = 0.23, *p* = 0.63
Dyslipidemia, *n* (%)	22 (79%)	14 (88%)	8 (67%)	χ^2^ = 3.22, *p* = 0.073
Smoking, *n* (%)	9 (32%)	5 (31%)	4 (33%)	χ^2^ = 0.44, *p* = 0.51
Chronic kidney disease, *n* (%)	12 (43%)	8 (50%)	4 (33%)	χ^2^ = 1.85, *p* = 0.17
Contrast agent,	75 (30; 200)	88 (30; 200)	65 (50; 200)	*p* = 0.85
Operation duration, min	95 (40; 240)	90 (40; 130)	100 (45; 145)	*p* = 0.36
Radiofrequency bursts, number	12 (6; 40)	12 (6; 40)	12 (9; 20)	*p* = 1.00
Hospitalization duration, bed-days	4 (3; 6)	4 (3; 6)	4 (3; 6)	*p* = 0.93
Office heart rate, bpm	71 (56; 98)	67 (56; 98)	75 (58; 88)	*p* = 0.96
Office SBP, mmHg	165 (125; 221)	169 (125; 221)	160 (140; 178)	*p* = 0.23
Office DBP, mmHg	100 (71; 138)	100 (71; 138)	100 (82; 116)	*p* = 0.49
Office pulse BP, mmHg	65 (50; 117)	68 (50; 117)	60 (51; 75)	*p* = 0.68
Mean 24 h SBP, mmHg	153 (104; 192)	146 (104; 192)	160 (127; 192)	*p* = 0.45
Mean 24 h DBP, mmHg	89 (62; 125)	79 (62; 125)	94 (69; 115)	*p* = 0.78
Mean daytime SBP, mmHg	157 (109; 202)	153 (109; 196)	163 (138; 202)	*p* = 0.49
Mean daytime DBP, mmHg	92 (67; 131)	88 (67; 131)	95 (76; 124)	*p* = 0.68
Mean nighttime SBP, mmHg	140 (99; 203)	135 (99; 203)	147 (109; 159)	*p* = 0.73
Mean nighttime DBP, mmHg	79 (54; 114)	71 (54; 114)	87 (55; 95)	*p* = 0.91
Non-dipping 24-h SBP profile, *n* (%)	11 (39%)	9 (56%)	2 (17%)	χ^2^ = 1.89, *p* = 0.17
Daytime BP load, %	81 (0; 100)	62 (0; 100)	94 (14; 100)	*p* = 0.30
Nighttime BP load, %	86 (0: 100)	69 (0; 100)	93 (13; 100)	*p* = 0.56
Creatinine, mcmol/l	74 (50; 192)	73.5 (56; 135)	75 (50; 95)	*p* = 0.93
Creatinine postoperative, mcmol/l	77 (47; 102)	76.5 (47; 102)	73 (48; 90)	*p* = 0.30
eGFR, ml/min/1.73 m^2^	89 (33; 114)	89.5 (53; 109)	94 (58; 114)	*p* = 0.64
Glucose, mmol/l	5.7 (4.6; 8.4)	5.6 (4.60; 8.40)	5.7 (5.00; 6.50)	*p* = 0.80
Total cholesterol, mmol/l	5.2 (3.9; 7.2)	5.2 (3.90; 7.10)	5.4 (4.2; 6.8)	*p* = 0.54
LDL-cholesterol, mmol/l	3.3 (1.2; 4.5)	3.3 (1.2; 4.5)	3.64 (3.2; 4.1)	*p* = 1.00
ESS, score	6 (3; 10)	5.5 (3; 7)	6 (3; 10)	*p* = 0.11

BMI – body mass index, BP – blood pressure, SBP – systolic blood pressure, DBP – diastolic blood pressure, ESS – Epworth sleepiness scale, eGFR – estimated glomerular filtration rate, LDL – low-density lipoprotein

Overall, patients were middle-aged, with the long-lasting HTN (over 10 years) and without other associated cardiovascular diseases. Every second patient was obese, every third patient had type 2 diabetes mellitus, and the majority of patients had dyslipidemia. Based on the initial ABPM data, all patients demonstrated stable HTN, none of them had masked or white-coat pattern.

Based on baseline PSG data, the patients were divided into two groups - with SDB (*n* = 16, AHI 17.3 (5.5;44.1)/h) and without SDB (*n* = 12, AHI 3.5 (1.0; 4.2)). The groups were matched by age, sex and important clinical parameters (Table 1). At baseline, in SDB group 4 patients showed severe SDB, 7 – moderate, and 5 – mild SDB. All cases were classified as obstructive sleep apnea (OSA). None of the patients used CPAP at the time of enrollment or during follow-up.

Although patients with SDB tended to be a bit older and to have dyslipidemia more frequently, the changes were not statistically significant. They also showed non-dipping pattern more frequently compared to non-SDB group (χ^2^ = 6.86, *p* = 0.009). The most frequently prescribed medications were RAAS blockers, calcium antagonists, centrally acting agents, and beta-blockers (Suppl. Table  2).

For RDN, Simplicity Flex™ catheter was used in 17 patients, Vessix™ Renal Denervation System – in 9 individuals, and Symplicity Spyral™ Renal Denervation system – in 2 subjects.

### Follow-up

The short-term results and adverse effects were described earlier [31] and included one linear dissection of renal artery and 1 femoral artery pseudoaneurysm which did not require any intervention.

In the present analysis, the average follow-up comprised 30.1 ± 8.4 months and was similar in SDB and non-SDB groups (31.1 ± 7.4 vs. 32.0 ± 6.0 months, *p* = 0.80).

The number of antihypertensive drugs decreased significantly (4.4 ± 1.1 vs. 3.8 ± 1.0, *p* = 0.028) which was mainly explained by the lower use of centrally acting medications (18 (64%) vs. 9 (32%), *p* = 0.021) (Suppl. Table  2).

**Fig. 2:**
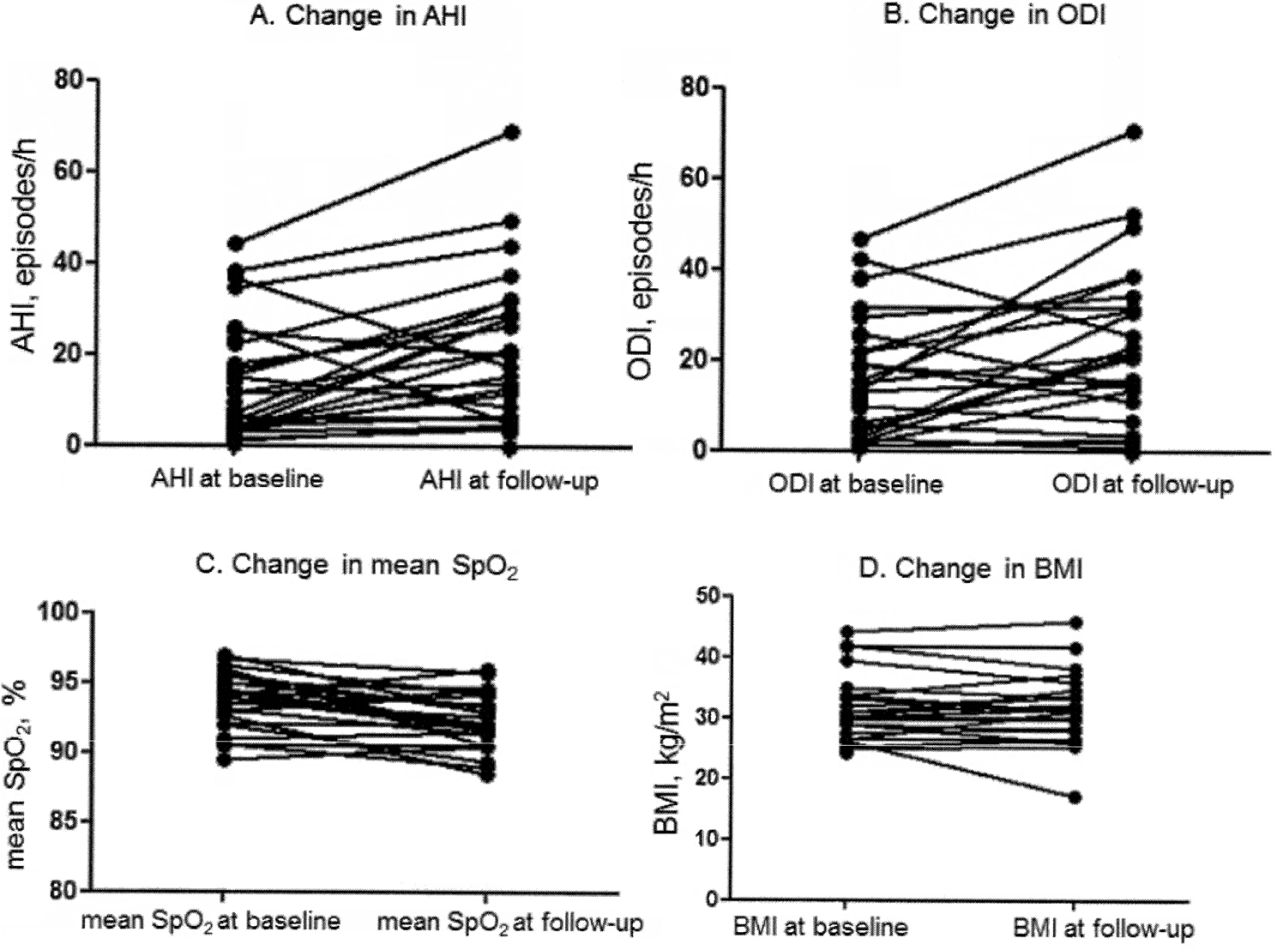
Changes in the indices of obstructive sleep apnea severity and body mass index

At long-term follow-up, no significant changes in creatinine level (*p* = 0.36) or eGFR (*p* = 0.074) were noted. There was no significant change in BMI from baseline (*p* = 0.79) (Table 2; Fig. 2).

**Table 2:** Patient characteristics at follow-up depending on the presence of baseline sleep-disordered breathing

Parameter at follow-up	Total (*n* = 28) median (min; max)	With SDB (*n* = 16)	Without SDB (*n* = 12)	*p*-value
BMI, kg/m^2^	31.5 (17.1; 45.8)	31.6 (17.1; 45.8)	31.2 (25.9; 37.1)	0.91
Number of antihypertensive drugs, *n*	4 (0; 6)	4 (0; 5)	4 (2; 6)	0.80
Creatinine, mcmol/l	83 (43.4; 139)	83 (43.4; 139.0)	72 (58.0; 99.9)	0.36
Δ Creatinine, mcmol/l	4 (-53; 30)	4 (-34; 22)	2.5 (-53; 30)	0.36
eGFR, ml/min/1.73 m^2^	79.5 (48; 124)	78 (48; 124)	96 (59.0; 109.0)	0.07
Δ eGFR, ml/min/1.73 m^2^	-5.98 (-31.2; 38)	-10.5 (-30.2; 36.0)	-0.2 (-22; 38)	0.36
Glucose, mmol/l	5.3 (4.3; 8.8)	5.2 (4.38; 8.80)	5.35 (4.30; 6.07)	0.73
Total cholesterol, mmol/l	4.9 (3.6; 8.3)	5.0 (4.0; 6.6)	4.8 (3.6; 7.1)	0.82
LDL-cholesterol, mmol/l	3.1 (1.5; 4.1)	3.2 (1.5; 4.1)	3.0 (2.4; 4.0)	0.68
Office heart rate, bpm	65 (51; 99)	63 (51; 87)	66 (60; 88)	0.49
Office SBP, mmHg	140 (110; 200)	140 (110; 200)	135 (120; 175)	0.60
Office DBP, mmHg	86 (65; 120)	88 (65; 120)	80 (70; 120)	0.78
Office pulse BP, mmHg	55 (40; 100)	56 (40; 100)	50 (43; 75)	0.60
Mean 24 h SBP, mmHg	138.5 (112; 192)	139 (112; 182)	137 (120; 192)	0.91
Mean 24 h DBP, mmHg	83 (66; 111)	83 (66; 105)	83 (72; 89)	0.69
Mean daytime SBP, mmHg	142 (114; 198)	140 (114; 181)	143 (125; 198)	0.80
Mean daytime DBP, mmHg	87 (69; 115)	87 (69; 107)	85 (75; 106)	0.69
Mean nighttime SBP, mmHg	135 (97; 187)	136 (97; 187)	116 (108; 173)	0.40
Mean nighttime DBP, mmHg	77 (56; 103)	80 (56; 102)	74 (61; 91)	0.59
**Non-dipping 24-h SBP profile,** ***n*** **(%)**	**14 (50%)**	**12 (75%)**	**2 (17%)**	**χ**^**2**^ **= 6.86,** *p*** = 0.009**
Daytime BP load, %	67 (5.5; 100)	58 (5.5; 100)	69 (21; 100)	0.79
Nighttime BP load, %	94 (0; 100)	94 (0; 100)	74 (24; 100)	0.64
ESS, score	6.5 (2.0; 14.0)	6.5 (2.0; 8.0)	5.0 (3.0; 14.0)	0.73

BMI – body mass index, BP – blood pressure, SBP – systolic blood pressure, DBP – diastolic blood pressure, ESS – Epworth sleepiness scale, eGFR – estimated glomerular filtration rate, LDL – low-density lipoprotein

### Changes in sleep-disordered breathing and sleep characteristics

Although the number of patients with verified SDB increased at follow-up (16 vs. 22), the SDB status (yes/no) changed insignificantly: one patient with baseline SDB demonstrated no OSA at follow-up and 3 patients without baseline SDB demonstrated AHI ≥ 5 episodes/h at follow-up (*p* = 0.13). All cases were classified as obstructive episodes. The distribution of the cases by OSA severity at baseline and follow-up was the following: severe (4 vs. 7 patients), moderate (7 vs. 8 patients), and mild (5 vs. 7 patients), respectively. Indices of SDB severity increased significantly, including AHI, oxygen desaturation index (ODI), mean and minimal O_2_ saturation (SpO_2_), hypoxia burden (Table 3; Fig. 2). The mean change in AHI comprised 9.0 (-21.1; 25.2) episodes/h indicating increase in SDB severity at follow-up mainly due to obstructive apneas (*p* = 0.939) and hypopneas index (*p* = 0.009) which were classified as obstructive ones. Both AHI in REM (rapid eye movement sleep) and NREM sleep increased. Similarly, the mean change in ODI was 6.5 (-16.8; 35.9) episodes/h. The average SpO_2_ showed a slight change by -1.7 (-5.6; 1.9)%. No correlation between the delta BMI and the delta AHI, ODI or average SpO_2_ was found (ρ = 0.25, *p* = 0.25; ρ = 0.09, *p* = 0.65, and ρ=-0.38, *p* = 0.07, respectively). Patients with positive and negative changes in AHI did not differ in the main clinical parameters either at baseline or at follow-up (data not presented).

**Table 3:** Changes in sleep-disordered breathing and sleep characteristics

	At baseline	At follow-up	*p*-value
SDB, *n* (%)	16 (57%)	22 (79%)	0.13
**AHI, episodes/h**	**11.4 (0.3; 44.1)**	**18.6 (0.0; 69.0)**	**0.005**
Obstructive apnea index, episodes/h	0.8 (0.0; 24.6)	2.4 (0.0; 43.1)	0.039
Central apnea index, episodes/h	0.0 (0.0; 8.4)	0.0 (0.0; 1.6)	0.75
Mixed apnea index, episodes/h	0.0 (0.0; 4.6)	0.2 (0.0; 10.6)	0.058
**Hypopnea index, episodes/h**	**7.8 (0.0; 27.6)**	**14.7 (0.0; 33.1)**	**0.009**
Mean apnea duration, sec	14.8 (0.0; 36.1)	15.3 (0.0; 36.7)	0.088
**AHI in NREM sleep, episodes/h**	**5.4 (0.0; 45.0)**	**17.1 (0.0; 67.8)**	**0.004**
**AHI in REM sleep, episodes/h**	**6.8 (0.0; 56.8)**	**26.1 (0.0; 73.9)**	**0.002**
AHI in supine position, episodes/h	19.6 (0.0; 96.1)	22.7 (0.0; 116.6)	0.15
**Desaturation index, episodes/h**	**12.7 (0.50; 46.7)**	**21.0 (0.0; 70.7)**	**0.012**
**SpO**_**2**_ **minimum, %**	**84.0 (67.0; 93.0)**	**81.0 (66.0; 91.0)**	**0.015**
**SpO**_**2**_ **average, %**	**94.2 (89.4; 97.0)**	**92.0 (88.5; 96.0)**	**0.002**
**Hypoxia burden (Time SpO**_**2**_ **< 90% of total sleep time), %**	**1.1 (0.0; 52.6)**	**7.5 (0.0; 67.1)**	**0.017**
**TST, min**	**417 (282; 516)**	**412.5 (180; 546)**	**0.018**
Sleep efficiency, %	82.3 (59.1; 93.6)	77.2 (27.4; 92.6)	0.70
WASO, min	57.7 (19.0; 204.5)	83.3 (11.5; 475.8)	0.28
Sleep latency, min	16.5 (1.5; 106.0)	21.0 (2.3; 95.0)	0.33
S1 sleep stage/TST, %	8.0 (3.2; 30.8)	8.9 (3.1; 41.1)	0.42
**S2 sleep stage/TST, %**	**47.5 (33; 69)**	**53.2 (32.5; 72.0)**	**0.007**
S3 sleep stage/TST, %	23.4 (0.0; 33.9)	16.8 (1.3; 53.1)	0.026
REM sleep stage/TST, %	20.3 (0.0; 29.5)	18.0 (0.0; 29.2)	0.17
Microarousal index, episodes/h	7.7 (0.0; 26.3)	13.2 (1.9; 42.4)	0.061
Awakenings, episodes	23 (14; 63)	26 (10; 62)	0.091
Limb movement index, episodes/h	7.5 (0.0; 105.2)	10.9 (0.0; 71.4)	0.13
Periodic limb movement index, episodes/h	7.6 (0.0; 88.4)	0.0 (0.0; 63.6)	0.86

SDB – sleep-disordered breathing, AHI – apnea-hypopnea index, REM sleep – rapid eye movement sleep, NREM sleep – non-rapid eye movement sleep, WASO – wake after sleep onset, TST – total sleep time

There were changes in sleep structure, including increase in the total duration of stage N2 and decrease in the total duration of stage N3.

### BP response

The short-term BP response in our cohort was described previously [31]. In present analysis we focused on the long- term (over 12-month) changes in BP and their association with the SDB severity. The BP (both in SBP and DBP) response did not differ in patients with and without SDB (Suppl. Table  3). The numbers of BP-responders were similar either when response was considered as at least 5-mmHg drop (Suppl. Table  3) or 10-mmHg drop (data not shown) in BP.

The number of non-dippers in SDB (baseline) group increased from 9 to 12 patients, while it remained the same in non-SDB group (2 vs. 2 patients, χ^2^ = 6.86, *p* = 0.009).

The baseline AHI, ODI and mean SpO_2_ showed weak correlations with the coefficient characterizing circadian systolic BP profile at follow-up (but not with baseline values): r = 0.51 (*p* = 0.019), r = 0.50 (*p* = 0.020), and r=-0.47 (*p* = 0.030), respectively. Regression analysis confirmed the association of circadian systolic BP profile status with the mean hypoxemia values SpO_2_ (OR -0.017 (-0.032; -0.001), *p* = 0.035), but not for AHI or ODI [AHI (OR 0.002 (0.001; 0.004), *p* = 0.10), ODI (OR 0.002 (-0.001; 0.004), *p* = 0.13)].

The follow-up indices of SDB severity did not show association with the circadian BP profile coefficients at follow-up visit. These indices did not correlate with the BP response either.

## Discussion

Our study demonstrated that at 3-year follow-up after RDN there is a worsening of OSA, i.e. an increase in the severity indices (AHI, ODI, hypoxemia burden). However, these changes are not associated with the BP response.

To our knowledge our study is the first to assess sleep parameters over 1 year after RDN. Our results do not support the previously suggested hypothesis of the beneficial effect of RDN on OSA. Since none of the previous studies has demonstrated a potential of RDN in OSA deterioration, we presume our patients experienced conventional progression of the disease.

On the other hand, the controversial results can be explained by the high variability in cohort characteristics in different studies. Thus, Shantha and Pancholy [32] performed a meta-analysis including 5 studies with rather small sample of 49 patients in total and stated a significant heterogeneity between the studies. The cohorts differ by OSA severity at baseline, the use of CPAP, severity of daytime sleepiness, comorbidity, the type of the RDN catheter system, etc. Despite the high heterogeneity, this meta-analysis demonstrated a reduction in AHI 6 months after RDN with the mean difference of -9.61/h (95% CI -15.43 to -3.79, *p* = 0.001), suggesting a significant improvement in OSA severity. Few studies reported improvement in other parameters of OSA severity, i.e. a decrease in daytime sleepiness assessed by Epworth sleepiness scale (ESS) [25], an increase in mean O_2_ saturation [26, 33, 34], a reduction in oxygen desaturation index (ODI) [33, 34].

Several studies enrolled CPAP-users [19, 25, 35], and succeeded in demonstrating benefit of RDN on top of CPAP-therapy. However, CPAP compliance data are not clearly reported, and, although baseline PSG was performed without CPAP, it is not clear whether the “washout” period was implemented in the PSG protocol for CPAP-users. Kiuchi et al. [19] in a controlled study (two arms - CPAP alone and CPAP + RDN) showed that RDN had a favorable impact on AHI even in patients with controlled HTN who demonstrated no antihypertensive benefit. Six months after the procedure, CPAP-users who underwent RDN demonstrated a greater reduction in AHI compared to control group (CPAP alone).

Intriguingly, a study which involved patients without CPAP-therapy did not confirm beneficial effects of RDN on OSA characteristics. Daniels et al. [36] did not find any significant change in any of the parameters of OSA severity 6 months post-RDN despite a significant BP response. They enrolled non-sleepy (ESS < 9 scores) patients with moderate-to-severe OSA (AHI ≥ 15/h) who had not been treated with CPAP. Although lack of daytime sleepiness was not an exclusion criteria in our study, our cohort was characterized by low ESS score (6 (3; 10)), and we might suggest that the low-symptomatic OSA (non-sleepy) could be less responsive to the treatment as it was shown with regard to the cardiovascular effects of CPAP-therapy [37, 38].

In our cohort, BP response at 3-year follow-up remained significant for both office systolic (*p* < 0.001) and diastolic BP (*p* = 0.012), but not for office heart rate, mean 24-hour, daytime and nighttime ABPM values. Unlike the data from the post-hoc analysis of the Symplicity HTN-3 study [35] and other studies [26], we did not find any between-group difference in BP response at long-term follow-up regarding either office BP or ABPM values. At the same time the number of non-dippers increased in the SDB group which correlates with the OSA severity indices, in particular, mean SpO_2_ values and corresponds to the well-known association between OSA and lack of nocturnal dipping [5]. The lack of nighttime BP response in our cohort can be explained by several factors. First, the OSA-related nocturnal BP elevation can be sustained in our cohort with persistent SDB despite RDN effect. Secondly, the reduction in antihypertensive therapy could consider mainly evening dosing leading to the lower control of nighttime BP. This is justified by the observations of acute BP response after RDN [39], and the common strategy to withdraw/reduce evening dose of antihypertensive medication. Although some authors suggest that evening dosing of certain antihypertensive drugs might be beneficial for 24-hour dipping BP profile, the recent large-scale populational studies have failed to prove its benefit regarding the major cardiovascular endpoints (the composite primary endpoint of vascular death or hospitalisation for non-fatal myocardial infarction or non-fatal stroke) [40], and the recent professional consensus does not recommend routine bedtime drug dosing [41]. However, based on our observations of the lack of post-RDN nighttime BP reduction in OSA patients we may speculate that this cohort might still benefit from bedtime antihypertensive dosing.

The evidence from Symplicity HTN-1, HTN-2 и HTN-3 [35] trials suggests that higher BP at baseline predicts a greater antihypertensive effect of RDN [11]. One could anticipate similar association for other effects of RDN, including the impact on OSA severity. However, available data from our study and other individual studies seem to be insufficient, and a pooled analysis including several cohorts is required. The clinical relevance of the observed changes in OSA severity is another issue which requires further analysis in a larger cohort and longer follow-up.

Another factor potentially associated with the RDN efficacy (for both BP values and OSA indices) is the type of the RDN catheter system. In our cohort, unipolar catheter Simplicity Flex™ was used in 17 patients, balloon technology multi-electrode catheter Vessix™ – in 9, and spiral catheter Symplicity Spyral™ – in 2 patients. So the majority of patients were operated by the unipolar system which might be of lower efficacy efficiency [11, 16]. The number of patients in each subgroup was insufficient to perform a between-group analysis. Moreover, one male patient operated with the use of Symplicity Spyral system showed a significant increase in AHI (from 5.5. to 28.1 episodes/h; no significant increase in BMI 27.4 versus 28.5 kg/m^2^), while the second one had severe OSA with the AHI 59/h remaining the same at follow-up. The exclusion of these patients from analysis did not affect the results. Various types of RDN catheter systems were used in published studies with no report on any differences in the impact on OSA severity to date.

The studies which support the beneficial effect of RDN on OSA severity suggest that RDN resulting in a decrease in sympathetic drive leads to a reduced extracellular fluid volume associated with the lower rostral fluid shift during sleep in recumbent position [25]. Moreover, RDN might be associated with the modulation of central sympathetic activity and its effects on respiratory center. We can speculate that these effects observed during the short-term follow-up attenuate with time, although antihypertensive effect of RDN is durable as shown by office BP values in our cohort and other studies [11, 22]. We cannot exclude that other factors could have an impact on AHI, ODI and other parameters of OSA severity. Among potential confounders aging does not seem to be significant at 3-year follow-up observation, and BMI remained stable in our cohort (*p* = 0.79). The changes in body composition and fat distribution could play a role [38], but we did not assess these parameters. The physiological variability in SDB should be taken into account. Although night-to-night AHI variability can be rather high [42], it rarely leads to an incorrect diagnosis or SDB severity evaluation [43], and some studies demonstrate no significant changes in apnea index over several years with the low predictive value of SDB severity measures at single time point [44]. In the prospective analysis from Sleep Heart Health Study, including 3040 subjects with repeated AHI assessment, the overall mean 5-year change in AHI was rather moderate and comprised 2.68 (10.58)/h, and the greater AHI change was associated with a larger BMI increase [45]. In contrast, in our cohort, BMI remained stable. To our knowledge, none of the RDN trials implemented multiple sleep studies at baseline or follow-up to exclude variability in OSA severity indices. In addition, although polysomnography was performed at the same sleep lab, the conditions could impact the results. At baseline the study was performed in in-patients before the RDN, while at follow-up out-patients were invited for the in-lab polysomnography.

Although we have not found a clear positive impact of RDN on OSA, the lack of BP elevation associated with the “natural” OSA progression may reflect a positive effect of RDN in this cohort of patients. Indeed, OSA progresses over time and in the lack of appropriate correction by CPAP therapy, one might expect the progression of cardiovascular regulation disturbance as well, resulting in non-response to the RDN procedure. Our findings contradict this assumption revealing the preserved effect of RDN on BP suppression despite the progression of OSA.

The strengths of our study include a comprehensive evaluation of the patients, and exclusion of secondary HTN, full in-lab PSG both at baseline and a follow-up scored by the same expert specialists, long-term follow-up of the cohort and the fact that none of the patients used CPAP-therapy either at baseline or during follow-up.

Our study has certain limitations which should be considered when interpreting the results. These include a non-randomized trial design, rather small sample size and loss to follow-up, the lack of short-term PSG assessment. The latter could result in missing short-term changes. The lack of a comparison to an untreated group is an important limitation, as we cannot exclude that AHI would have increased even more over 30 months in an untreated comparison cohort. However, as indicated earlier the worldwide data demonstrate no significant changes in apnea index (leading to a different diagnosis [SDB vs. no SDB] or severity evaluation) over up to 5 years [44, 45]. Nevertheless, taken a very limited data on the RDN effects on SDB characteristics, our study provides new important insights on the long-term outcomes of the RDN in OSA patients.

## Conclusion

At long-term (> 12 months) follow-up after renal denervation the severity of obstructive sleep apnea is not attenuated, but worsened with the increase in apnea-hypopnea and desaturation indices and the decrease in mean nocturnal oxygen saturation.

Although the severity of OSA worsens at > 12 months follow-up after RND, this is not associated with hypertension exaggeration. The potential clinical relevance of the observed changes in OSA severity and their association with the type of renal denervation system used require further assessment.

### Electronic supplementary material

Below is the link to the electronic supplementary material.

Supplementary Material 1: **Table 1**. Characteristics of the included and non-included patients. **Table 2**. Antihypertensive and concomitant therapy at baseline and follow-up. **Table 3**. Blood pressure response depending on sleep-disordered breathing presence at baseline

## Data Availability

The data that support the findings of this study are available on reasonable request from the corresponding author. The data are not publicly available due to privacy or ethical restrictions.
