# Supplementary material for: Progression of obstructive sleep apnoea after renal denervation is not associated with hypertension exaggeration
Source: BMC Pulm Med. 2023 Nov 23;23:467. doi: 10.1186/s12890-023-02757-1 (PMC10668416; doi:10.1186/s12890-023-02757-1)
Supplement: Supplementary file 1 — Supplementary Material 1: Table 1. Characteristics of the included and non-included patients. Table 2. Antihypertensive and concomitant therapy at baseline and follow-up. Table 3. Blood pressure response depending on sleep-disordered breathing presence at baseline [file 12890_2023_2757_MOESM1_ESM.docx]

Supplemental table 1. Characteristics of the included and non-included patients

| Parameter  [n (%) or Me (Min; Max)] | Included patients (with available sleep studies at both baseline and follow-up)  N=28 | Non-included patients (without sleep studies at baseline and at follow-up)  N=42 | p-value |
| --- | --- | --- | --- |
| Sex (male), n (%) | 15 (53%) | 14 (33%) | χ^2^=2.84, p=0.09 |
| Age, years | 54.5 (27; 69) | 55 (30; 80) | p=0.68 |
| HTN duration, years | 16.5 (4; 36) | 15 (1; 50) | p=0.35 |
| Antihypertensive drugs, number | 4 (2; 7) | 4.5 (2; 7) | p=0.55 |
| BMI, kg/m^2^ | 30.0 (24.2; 44.2) | 31.2 (18.8; 38.3) | p=0.70 |
| SBP, mmHg | 165 (125; 221) | 153 (140; 188) | p=0.004 |
| DBP, mmHg | 100 (71; 138) | 90 (69; 110) | p=0.014 |
| Heart rate, bpm | 71 (56; 98) | 71.5 (54; 92) | p=0.91 |
| Mean 24h SBP, mmHg | 153 (104; 192) | 142 (106; 227) | p=0.24 |
| Mean 24h DBP, mmHg | 89 (62; 125) | 79 (53; 118) | p=0.13 |
| Mean daytime SBP, mmHg | 157 (109; 202) | 147 (113; 227) | p=0.15 |
| Mean daytime DBP, mmHg | 92 (67; 131) | 86 (52; 118) | p=0.10 |
| Mean nighttime SBP, mmHg | 140 (99; 203) | 135 (94; 227) | p=0.55 |
| Mean nighttime DBP, mmHg | 79 (54; 114) | 73 (46; 117) | p=0.26 |
| eGFR, ml/min/1.73 m^2^ | 89 (33; 114) | 86 (40; 119) | p=0.10 |
| Type 2 diabetes mellitus, n (%) | 7 (25%) | 14 (33%) | χ^2^=0.56, p=0.46 |
| Dyslipidemia, n (%) | 22 (79%) | 28 (67%) | χ^2^=1.17, p=0.28 |
| Smoking, n (%) | 9 (32%) | 10 (24%) | χ^2^=0.59, p=0.44 |
| CKD, n (%) | 12 (42%) | 12 (29%) | χ^2^=1.52, p=0.22 |
| Office BP response, n (%) | 19 (68%) | 23 (55%) | χ^2^=1.20, p=0.27 |

BMI – body mass index, BP – blood pressure, SBP – systolic blood pressure, DBP – diastolic blood pressure, eGFR – estimated glomerular filtration rate, CKD – chronic kidney disease

Supplemental table 2. Antihypertensive and concomitant therapy at baseline and follow-up

| Medication | Baseline | Follow-up | p-value |
| --- | --- | --- | --- |
| Number of antihypertensive drugs | 4.4 (2; 7) | 3.8 (2; 6) | 0.028 |
| RAAS blocker, n (%) | 26 (93%) | 24 (86%) | 1.00 |
| Beta-blocker, n (%) | 16 (57%) | 19 (68%) | 0.25 |
| Calcium channel blocker, n (%) | 25 (89%) | 22 (79%) | 0.73 |
| Thiazide diuretic, n (%) | 19 (68%) | 21 (75%) | 0.63 |
| Loop diuretic, n (%) | 8 (29%) | 6 (21%) | 1.00 |
| Aldosterone antagonist, n (%) | 5 (18%) | 5 (18%) | 1.00 |
| **Central action medication, n (%)** | **18 (64%)** | **9 (32%)** | **0.021** |
| Statin, n (%) | 16 (57%) | 15 (54%) | 1.00 |

RAAS – renin-angiotensin-aldosterone system

Supplemental table 3. Blood pressure response depending on sleep-disordered breathing presence at baseline

| Parameter | N | Total (n=28) | With SDB (n=16) | Without SDB (n=12) | p-value |
| --- | --- | --- | --- | --- | --- |
| Change in BP at discharge | | | | | |
| Office SBP at discharge, mm Hg | 28 | 136 (108; 170) | 140 (108; 170) | 121 (115; 159) | p=0.24 |
| Δ office SBP at discharge, mmHg | 28 | -33 (-80; 19) | -34 (-80; 19) | -37.5 (-57; 19) | p=1.00 |
| Office DBP at discharge, mm Hg | 28 | 80 (54; 106) | 82 (54; 106) | 80 (70; 100) | p=0.36 |
| Δ office DBP at discharge, mmHg | 28 | -18.5 (-56; 24) | -20 (-56; 24) | -18.5 (-36; -1) | p=1.00 |
| Change in BP at 12-36-month follow-up | | | | | |
| Δ office SBP at follow-up, mmHg | 28 | -19.5 (-93; 20) | -23 (-93; 20) | -15 (-43; 5) | p=0.76 |
| Δ office DBP at follow-up, mmHg | 28 | -11 (-59; 20) | -11.5 (-59; 10) | -11 (-35; 20) | p=0.72 |
| Δ office pulse BP at follow-up, mmHg | 28 | -7.5 (-40; 25) | -7 (-40; 25) | -8 (-25; 10) | p=0.93 |
| Δ office SBP≥5 mmHg at follow-up, | 28 | 21 (75%) | 13 (81%) | 6 (50%) | χ^2^=0.67, p=0.41 |
| Δ office DBP≥5 mmHg | 28 | 18 (64%) | 11 (69%) | 6 (50%) | χ^2^=0.01, p=0.92 |
| Δ office pulse BP≥5 mmHg | 28 | 16 (57%) | 9 (56%) | 6 (50%) | χ^2^=0.26, p=0.61 |
| Δ 24h SBP, mmHg | 22 | -2.5 (-70; 73) | -4.5 (-70; 73) | -1.5 (-33; 25) | p=1.00 |
| Δ 24h SBP≥5 mmHg | 22 | 10 (36%) | 7 (44%) | 2 (17%) | χ^2^=0.47, p=0.49 |
| Δ 24h DBP, mmHg | 22 | -3 (-37; 38) | -3 (-37; 38) | -13 (-32; 9) | p=0.60 |
| Δ 24h DBP≥5 mmHg | 22 | 11 (39%) | 7 (44%) | 4 (34%) | χ^2^=0.47, p=0.49 |
| Δ daytime SBP, mmHg | 22 | -3.5 (-71; 70) | -7.5 (-71; 70) | -2.5 (-30; 22) | p=1.00 |
| Δ daytime SBP≥5 mmHg | 22 | 10 (36%) | 7 (44%) | 2 (17%) | χ^2^=0.47, p=0.49 |
| Δ daytime DBP, mmHg | 22 | -3.0 (-43; 34) | -5 (-43; 34) | -10 (-30; 7) | p=0.72 |
| Δ daytime DBP≥5 mmHg | 22 | 10 (36%) | 7 (44%) | 3 (25%) | χ^2^=0.01, p=1.00 |
| Δ nighttime SBP, mmHg | 22 | 2 (-73; 73) | 4 (-73; 73) | 2 (-37; 40) | p=0.84 |
| Δ nighttime SBP≥5 mmHg | 22 | 9 (32%) | 6 (38%) | 2 (17%) | χ^2^=0.16, p=0.69 |
| Δ nighttime DBP, mmHg | 22 | 3 (-33; 70) | 3 (-33; 70) | -2.5 (-28; 18) | p=0.55 |
| Δ nighttime DBP≥5 mmHg | 22 | 10 (36%) | 6 (38%) | 3 (25%) | χ^2^=0.09, p=0.77 |

SBP – systolic blood pressure, DBP – diastolic blood pressure
